# Transcriptomics of Parental Care in the Hypothalamic–Septal Region of Female Zebra Finch Brain

**DOI:** 10.3390/ijms23052518

**Published:** 2022-02-24

**Authors:** Rashmi Kumari, Emese A. Fazekas, Boglárka Morvai, Edina B. Udvari, Fanni Dóra, Gergely Zachar, Tamás Székely, Ákos Pogány, Árpád Dobolyi

**Affiliations:** 1 MTA-ELTE Laboratory of Molecular and Systems Neurobiology, Eötvös Loránd Network of Research Excellence and Eötvös Loránd University, 1117 Budapest, Hungary; rashmi.kaul17@gmail.com (R.K.); fazeme19@gmail.com (E.A.F.); edina328@gmail.com (E.B.U.); 2 Department of Physiology and Neurobiology, Eötvös Loránd University, 1117 Budapest, Hungary; 3 Department of Ethology, Eötvös Loránd University, 1117 Budapest, Hungary; boglarka.morvai@ttk.elte.hu (B.M.); akos.pogany@ttk.elte.hu (Á.P.); 4 Department of Anatomy, Histology and Embryology, Semmelweis University, 1093 Budapest, Hungary; fanni.dora@gmail.com (F.D.); gzachar@gmail.com (G.Z.); 5 Milner Centre for Evolution, Department of Biology and Biochemistry, University of Bath, Bath BA2 7AY, UK; bssts@bath.ac.uk

**Keywords:** parental care, transcriptome sequencing, RNA-Seq, gene expression, gene ontology, social behaviour, hypothalamic–septal region, posthatching period, zebra finch mother

## Abstract

(1) Background: The objective of this study was to uncover genomic causes of parental care. Since birds do not lactate and, therefore, do not show the gene expressional changes required for lactation, we investigate gene expression associated with parenting in caring and non-caring females in an avian species, the small passerine bird zebra finch (*Taeniopygia guttata*). Here, we compare expression patterns in the hypothalamic–septal region since, previously, we showed that this area is activated in parenting females. (2) Methods: Transcriptome sequencing was first applied in a dissected part of the zebra finch brain related to taking care of the nestlings as compared to a control group of social pairs without nestlings. (3) Results: We found genes differentially expressed between caring and non-caring females. When introducing a log2fold change threshold of 1.5, 13 annotated genes were significantly upregulated in breeding pairs, while 39 annotated genes were downregulated. Significant enrichments of dopamine and acetylcholine biosynthetic processes were identified among upregulated pathways, while pro-opiomelanocortin and thyroid hormone pathways were downregulated, suggesting the importance of these systems in parental care. Network analysis further suggested neuro-immunological changes in mothers. (4) Conclusions: The results confirm the roles of several hypothesized major pathways in parental care, whereas novel pathways are also proposed.

## 1. Introduction

Parental behaviour is adaptive as it increases the survival chance of the offspring, thereby contributing to fitness [1,2]. Due to its importance, parental behaviour is a frequently investigated form of social behaviour. The genome and parental behaviour are closely related and can influence each other through complex regulatory mechanisms [3]. Several brain regions known to play a role in the control of maternal behaviours have been investigated for gene expressional changes related to parental care in recent years in rodents. Using the microarray technique, altered gene expression was found in the medial preoptic area [4], in the septum [5] and in the nucleus accumbens during the postpartum maternal state [6]. These microarray analyses identified a large subset of genes participating in neuronal processes, e.g., in ion channel activity and neuronal development [4]. Similar microarray approaches were also performed in the medial preoptic area (MPOA) of postpartum rat dams and identified a few genes related to maternal adaptation and induction of maternal care [7,8]. The more recently developed alternative method for gene expression analysis, RNA-sequencing [9,10], has also been used to study parental gene expressional changes in different species including mice [11] and rat [12]. In birds, RNA-sequencing has been performed in non-reproductive Japanese quail [13], in tree swallows and Muscovy ducks to address neurogenomic changes associated with incubation [14,15], and in blackbirds to understand the loss of maternal care in Avian brood parasites [16]. In addition, proteomics approaches have also been used in mammals to compare protein levels between maternal and non-maternal females. These studies revealed that protein levels differ in the medial prefrontal cortex [17], the hypothalamus [18], and the preoptic area [19] depending on the status of the female.

Correlating gene expression levels and their molecular function with the expression of behaviour is still challenging, due to the complexity of the regulation of behaviours and the accompanying physiological changes [3]. In mammals, physiological changes in mothers are especially complex because they have to support lactation. During lactation, hormonal changes maintain pup-induced milk production as well as increased food and fluid intake required for milk synthesis [20]. To eliminate the confounding contribution of lactation-related events to the gene expressional changes, we selected an avian species, the zebra finch (*Taeniopygia guttata*). While some avian species, including pigeons, produce crop milk [21], zebra finches feed their offspring via regurgitation without crop milk production [22]. To account for reproductive-stage-dependent and diurnal changes of parenting, behavioural and tissue sample collection was always carried at the same time point and reproductive stage—on the 10th day of the post-hatching period to assess parental behaviour of the animals—while dissections of brains were performed on the 13th day (PHD-10 and PHD-13, respectively), when caring behaviour is very intense. Zebra finches are biparental species, but females spend more time parenting than males [23]. Therefore, females were examined in the present study. Gene expressional alterations can be caused by adult social interactions too [24,25]. Therefore, to reduce the contribution of adult social interactions to the gene expressional changes, we chose social pairs without offspring as negative controls. Female zebra finches do not necessarily accept any offered males paired by the researchers for mating. Rather, they select males, based primarily on songs and morphological traits, with whom they form social pairs and mate [26]. However, without nesting opportunity, the male and female remain a social pair but will not have offspring. By using social pairs without nest material as the control group in the present gene expression study, potential social pairing- and social interaction-evoked gene expressional changes would be similar in both the experimental and control groups so that the differentially expressed genes would be predominantly related to parental behaviours.

Gene expressional changes related to parental behaviours likely occur in brain regions involved in the control of caring behaviour. The parental brain network is relatively well-known in mammals [27]. The preoptic area of the hypothalamus is the most important brain region involved in the control of parental behaviours [28,29,30]. Other hypothalamic brain regions are important in specific aspects of parenting too, while adjacent limbic areas, including the septum, may also be involved in parenting [31]. In a previous study, we found that nestling-induced c-fos activation pattern in the brain of the zebra finch is similar to that in mammals [32]. Most of the activated brain regions were found in the septal–hypothalamic region of the brain [32] as previously established in response to pup-induced c-fos activation studies in rodents [33,34]. Based on this finding, we assumed that the septal–hypothalamic region of the bird brain is the most significant area in the control of parental behaviours. Therefore, this brain region was dissected in the present study. The specific objectives were (i) to investigate the genetic and molecular background of parental behaviours in female zebra finches compared to socially paired females with RNA-Seq gene expression analysis, and (ii) to identify molecular pathways associated with parenting. Determining the genetic and molecular background of parental behaviours would lead us to understand how this complex behaviour is regulated in zebra finches, where parenting-related gene expressional changes have not been addressed yet. This transcriptomic analysis may also provide the background of further comparative evolutionary studies to investigate how distinct forms of parenting strategies evolved in different species.

## 2. Results

### 2.1. RNA Sequencing

After cleaning and filtering, the number of reads retained per sample was >21,000,000. The STAR2 overall alignment rates exceeded 82% for each sample (Table 1). A total of 31,622 genes were expressed in all the samples; among them, 17,875 were well-known annotated genes.

### 2.2. Differential Gene Expression Analysis

**Figure 1:**
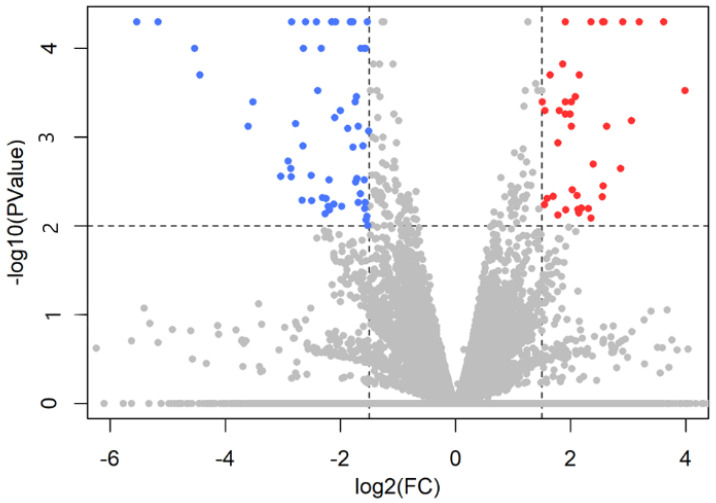
The results of sequencing shown in a volcano plot. Significantly upregulated genes are red in the upper right corner, while significantly downregulated genes are blue in the upper left corner. Because the test is now based on explicit sampling from the beta negative binomial in Cuffdiff v.2.2.1., users do not see values less than 10^−5^ by default.

The distribution of *p* value of the genes as a function of fold change is shown in a volcano plot (Figure 1). There were 52 differentially expressed genes (DEGs) identified in the hypothalamic–septal regions between the breeding and paired groups. In the breeding group, 13 genes were upregulated and 39 genes were downregulated compared to the paired group. The threshold of *p* value was 0.05 to filter the significant DEGs, and the threshold of log2FC value was ±1.5 to define up- and downregulated genes. Among 13 upregulated and 39 downregulated genes, 7 and 24 were well-annotated, respectively (Table 2).

### 2.3. Validation

**Figure 2:**
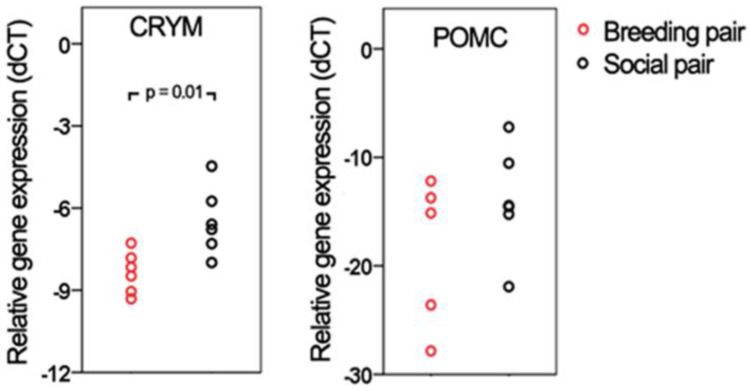
The results of PCR validation. The CRYM showed a significant difference between breeding and social pairs (*p* < 0.01), while POMC demonstrated a strong tendency (*p* < 0.1). The red dots represent individual females of breeding pairs, while black dots are individual females of social pairs.

First, the expression level of housekeeping genes was examined in all samples (*n* = 12) and normal distribution was found (Shapiro–Wilk test, *p* > 0.05). Because of the limited amount of cDNA available, only two DEGs were used to validate the sequencing results with qRT-PCR, CRYM and POMC. The distribution of the expression levels was checked for both selected genes and normal distribution was found (Shapiro–Wilk test, *p* > 0.05). For POMC, the sample size was reduced to five due to loss of signal in PCR reactions. CRYM showed significantly different gene expression based on qRT-PCR as it was downregulated in the breeding group (*p* < 0.01), while POMC showed a strong tendency (*p* < 0.1; Figure 2) with a similar fold change (−4.51 for PCR and −5.52 for sequencing).

### 2.4. Parental Behaviour of Breeding Pairs

Parental behaviour was analysed from the 3 h long observations recorded on PHD-10. The interaction with the mate was also observed, whereas the males’ behaviour was only registered as presence or absence. Females spent 53.85% of the total time (180 min) with parenting, while the males spent 35.37% of their time in the nest. The time they spent together was only 5.55% of the total time.

### 2.5. Correlation of Behaviour with Gene Expression

**Figure 3:**
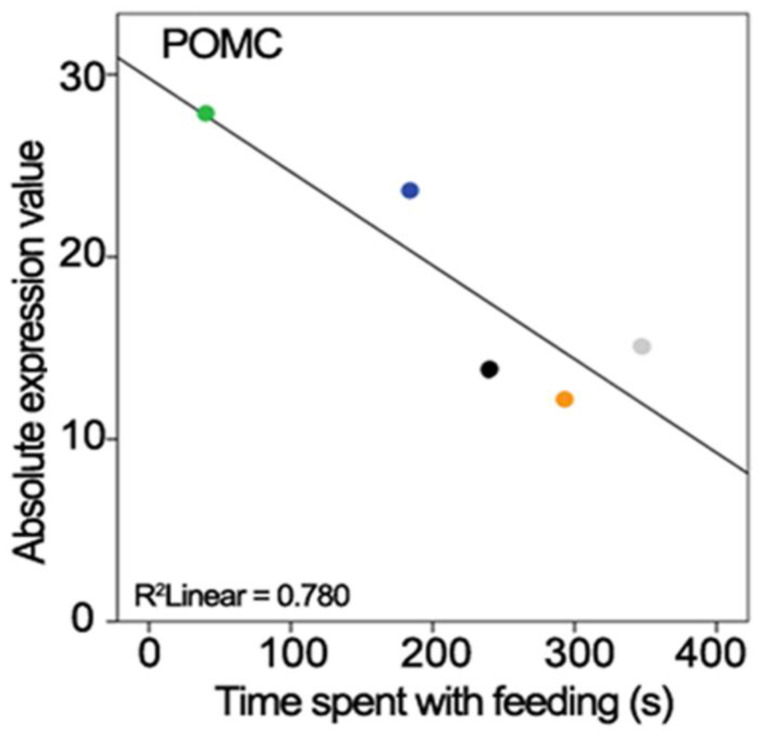
Correlation between gene expression levels and the time spent parenting in zebra finches. Panel shows the negative correlation of POMC gene expression level and time of feeding behaviour. The coloured dots represent the individuals.

The gene expression level measured with PCR and the performed behaviours were correlated. The time spent feeding the offspring was negatively correlated with the POMC gene expression level (r = −0.883, *n* = 5, *p* < 0.05; Figure 3).

### 2.6. Relationship of DEGs

**Figure 4:**
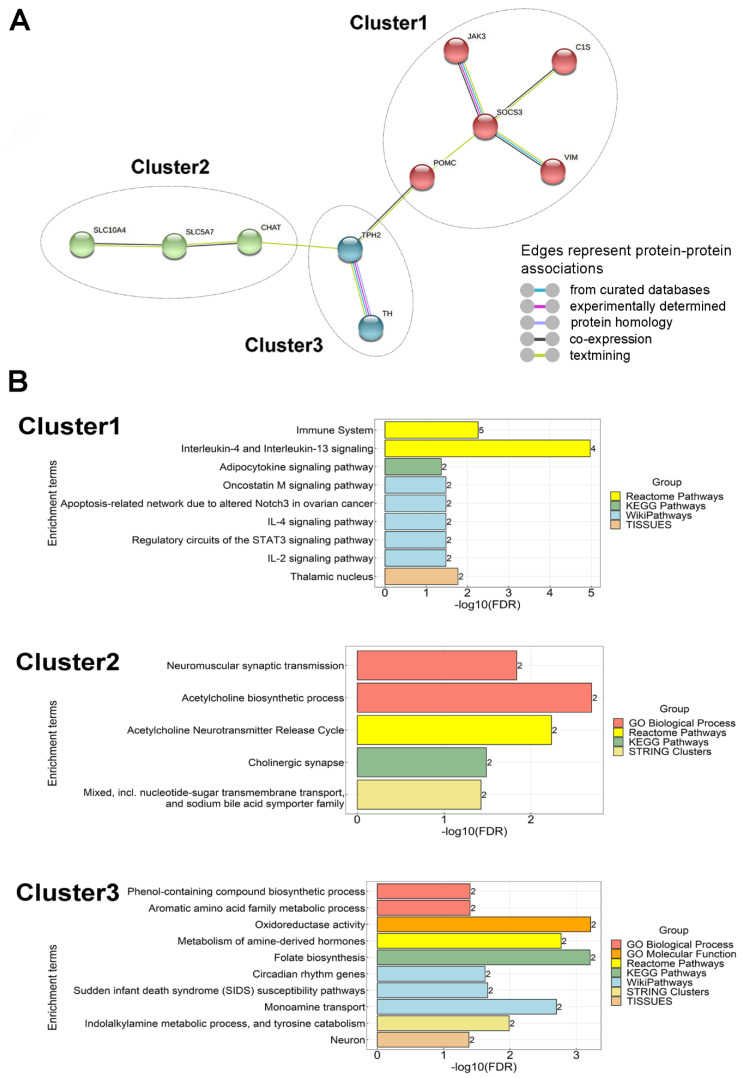
Interactions between proteins including down- and upregulated genes, as predicted by the STRING online database. (**A**) PPI network of 5 down- and 5 upregulated genes was constructed. The colour of the nodes represents the three clusters. Each edge colour indicates a different method of protein–protein association prediction as indicated in the figure. The enrichment *p* value of PPI network for the number of identified edges compared to the expected (2) was 0.000575, significantly more than expected with a medium interaction score of 0.4. Clustering PPI network with Markov Clustering algorithm (MCL) shows three clusters of DEGs. Cluster 1 represents the protein–protein association of downregulated genes, while Clusters 2 and 3 include the functionally connected upregulated genes. (**B**) Clusters were functionally annotated. Gene Ontology (GO) and pathway classifications of the three clusters of PPI network were analysed through the STRING online database with the FDR < 0.05. Bar graphs show the enriched terms of Cluster 1 including 5 downregulated genes, and Clusters 2 and 3 including 5 upregulated genes. In the graph, *y*-axis represents the significantly enriched terms. Each bar describes the number of mapped annotated genes in the reference dataset, while the *x*-axis indicates the significance (−log10 (FDR)). The colour code corresponds to the ontologies.

To determine the functional significance of our data, a protein–protein interaction analysis of DEGs was performed. It revealed that there were three functional clusters. Interestingly, one cluster contained only downregulated genes and two only upregulated genes. There were nine interactions represented by the edges (lines) between each node (DEGs), suggesting that these genes are biologically connected (Figure 4A). The functions of downregulated genes in Cluster 1 with the highest levels of enrichment included roles such as interleukin-4 and interleukin-13 signalling and regulatory circuits of the STAT3 signalling pathway in the immune system (Figure 4B). Additional functional enrichment analysis identified significant differences in pathways of upregulated genes. Cluster 2 was significantly enriched in the acetylcholine neurotransmitter release cycle, while Cluster 3 was enriched in monoamine metabolism (Figure 4B).

### 2.7. Gene Ontology Analysis

The functions of up- and downregulated genes were accessed using gProfiler GO analysis with the alpha significance threshold of 0.05. The KEGG functional categorisation and the gene set enrichments analysis were performed separately for both the up- and downregulated genes. We identified a number of significantly altered KEGG pathways and gene ontologies, among which the most significant ones are shown in Table 3. As individual DEGs have been identified (see above), here, we instead focus on genes, which contributed to a KEGG pathway or gene ontology together with another gene or genes in any category. These eight upregulated and seven downregulated genes are listed in Table 4. Most of these genes are DEGs by definition and are presented in Table 2. Listing these DEGs in Table 4 indicates that these (and not other) DEGs are actually part of altered pathways. In addition, some other genes listed in Table 4, which were changed significantly but without reaching the fold change limit, also contribute to significantly altered pathways. For example, vesicular monoamine and vesicular acetyl-choline transporters are not DEGs but their significant change still supports that dopaminergic and acetylcholinergic synaptic transmission was elevated in the maternal brain.

## 3. Discussion

### 3.1. Gene Expressional Studies in the Zebra Finch

In avian species, brain tissue was used mostly in comparative studies that investigated full brain transcriptomics [35,36]. Recently, experiments comparing gene expression in different zebra finch brain parts were successfully performed [37], showing promise for use of the technique to study selected brain regions as we did in our study. In this sense, our study is also comparable to previous microarray studies performed in zebra finch brain. These identified a different gene expression pattern in vocal brain centres and surrounding brain regions [38,39]. As RNA-Seq studies in birds are still emerging, we will first discuss the interpretation and reliability of our sequencing data. Then, DEGs and gene sets demonstrated to be enriched in the up- or downregulated genes and pathways suggested to be altered will be considered. Finally, a larger picture of how parental adaptation takes place in the brain of zebra finch and how it relates to mammalian maternal gene expressional changes will be discussed.

### 3.2. Methodological Considerations

The brain area dissected in the study includes hypothalamic and septal regions, which are known to control maternal behaviours in mammals [20] and which have been demonstrated to contain activated neurons in response to exposure to the nestling in the zebra finch [32]. Still, a relatively large brain area was dissected, and the demonstrated gene expressional changes can take place in all the cells, from which mRNA was isolated. Therefore, if a gene is found to be differentially expressed, it is the sum of changes in individual cells, so either the gene expression level changes in all the cells or it changes dramatically in some cells and does not change in others. Neurons, which were activated in zebra finch mothers in response to their nestlings, are expected to change the expression of some of their genes in the presence of nestlings but they represented only a small subset of neurons even within the hypothalamic–septal brain region [32]. Other cells may also demonstrate gene expressional changes in the post-hatching period; still, many of the cells may be non-responsive and may mask the alterations. Thus, only the markedly altered gene expressional changes can be detected.

The above consideration requires a reliable measurement to detect possibly small gene expressional alterations. In our sequencing, the number of reads per sample was very high, comparable to other recent high quality sequencing from zebra finch brain [37]. The percentage of mapped reads was also high, and importantly, similar between the breeding and paired control groups of animals. Finally, the identified 17,875 known genes in the samples also suggested that a reliable sequencing was performed.

The total number of DEGs, 52, is similar to studies using mammalian models with similarly large fold change limits (logFC = 1.5) [12]. Some of the DEGs were up-, others downregulated. The two directions were not fully symmetrical as more down- than upregulated genes were found. Since we have no reason to presume any methodological bias, we assume that more genes decrease than how many increase their expression level during the post-hatching period. It also has to be noted that the expression levels of the vast majority of genes did not change at all during this period.

Our knowledge on the mechanisms of how gene expressions are altered in mothers is limited. An intriguing possibility is regulation by transposable elements, which can carry potent transcriptional cis-regulators that could recruit specific transcription factors, even in a tissue-specific manner [40,41]. There is available evidence of the involvement of transposable elements insertions in gene expression in the brain [42,43,44] and their role in the zebra finch brain is also plausible.

### 3.3. The Possible Involvement of Gene Sets and Pathways in Maternal Adaptation and Behaviour

#### 3.3.1. Dopaminergic System

Tyrosine hydroxylase enzyme (TH) catalyses the rate-limiting conversion of tyrosine to L-DOPA, which is a precursor of dopamine as well as norepinephrine and epinephrine [45]. Therefore, the elevated expression of TH gene may refer to the increased need of catecholamines during breeding. While this is the first report of increased TH level in relation to parenting in birds, the induction of TH has been reported during courtship behaviour in songbirds [46], suggesting the availability of the machinery needed for its induction in relation to social behaviour. Furthermore, elevated TH level was also found in rodents in the postpartum period [47].

Another upregulated gene that is involved in the dopamine system is SLC6A2 (solute carrier family 6 member 2), which encodes a transporter of the sodium:neurotransmitter symporter family. It regulates the reuptake of dopamine and norepinephrine into the presynaptic terminal of synapses [48]. In birds, SLC6A2 is expressed in dopaminergic cells too, as avian species lack the dopamine transporter gene (SLC6A3/DAT), so SLC6A2 serves the function of DAT [49]. In addition, neuronal cell bodies using norepinephrine and epinephrine as neurotransmitters are not present in this region, also supporting that the elevated SLC6A2 implies increased reuptake of dopamine. Thus, our study is the first to report that SLC6A2 is upregulated in the hypothalamic–septal area of breeding females, suggesting the increased reuptake of dopamine.

Genes responsible for vesicular monoamine transport were also upregulated. Solute carrier family 10 member A4 (SLC10A4), responsible for acidification of synaptic vesicles containing monoamines [50], was itself significantly upregulated while solute carrier family 18 member A2 (SLC18A2), the synaptic vesicular monoamine transporter, was found among the genes responsible for the regulation of neurotransmitter levels in the synapse GO terms.

As discussed above, several elements of the dopaminergic system were upregulated, providing strong evidence of their increased activity in the breeding period. There are different dopaminergic cell populations located in the dissected brain region, and the current data provide no evidence of which one had increased activity. Some neurons of the ventral tegmental area may have been included in our dissections. Its dopaminergic neurons are involved in the reward system, which is certainly a candidate to play a part in caring behaviour as the pups in mammals but also the nestlings in birds represent a reward for their mother [51]. Another dopaminergic cell group located in the hypothalamic arcuate nucleus regulates prolactin release [52]. Prolactin is important in lactation but also in maternal behaviours in mammals [53]. In birds, prolactin promotes brooding behaviour and possibly also feeding the young [54]. However, dopamine negatively regulates prolactin release [55]. Therefore, the activity of dopaminergic neurons regulating prolactin secretion from the pituitary is actually reduced while taking care of the young, making it unlikely that the upregulation of dopaminergic function took place in this cell group. However, an additional dopaminergic cell group of the hypothalamus was recently suggested to promote maternal behaviours in mice [56]. These cells located in the periventricular zone of the hypothalamus likely have corresponding avian counterparts, which may also increase their activity to promote caring behaviour.

#### 3.3.2. Serotonergic System

The TPH2 gene encodes an enzyme (tryptophan hydroxylase 2) that has tryptophan 5-monooxygenase activity and is involved in the synthesis of serotonin in the central nervous system [57]. The TPH2 enzyme is dominantly expressed in the raphe nuclei of which the midbrain dorsal raphe was involved in the dissected brain region. Neuroplastic changes in these serotonergic neurons by infant contact have been reported during motherhood [58]. Serotonin is involved in diverse neural functions, such as sleep, aggression, food intake, and sexual behaviour [59]. Our data suggest that serotonin may also contribute to the changes and adaptation mechanisms of parenting.

#### 3.3.3. Cholinergic System

A gene upregulated in the study was CHAT, which encodes the enzyme choline-O-acetyltransferase. This enzyme catalyses the biosynthesis of the neurotransmitter acetylcholine at cholinergic presynaptic terminals. The upregulation of CHAT can be indicative of elevated acetylcholine level in breeding zebra finches.

The SLC5A7 (solute carrier family 5 member 7) gene encodes a transporter (ion and chloride ion-dependent high-affinity transporter) that mediates the reuptake of choline to the presynaptic terminal of cholinergic neurons for the synthesis of acetylcholine [60].

Strong evidence was provided by our study on the upregulation of cholinergic neurons in the female parent during the post-hatching period, as elevated expression was found for both CHAT and SLC5A7. The induced level of the synthesizing enzyme of acetylcholine and also of the choline transporter suggest enhanced cholinergic neurotransmission, implying increased activity of cholinergic cells during breeding. The cholinergic system has been suggested to be a regulator of parental behaviour in mice, because inhibition of muscarinic acetylcholine receptors disturbed the caring behaviour [61]. In birds, this is the first report to implicate the cholinergic system in parental behaviour as, previously, only vocalization has been mentioned as a reproductive social behaviour involving the cholinergic system [62]. Cholinergic neurons within the dissected brain regions may be located in the medial septum, the posterior part of the accumbens nucleus and some parts of the basal forebrain. Acetylcholine in the accumbens nucleus released from local interneurons acting on dopaminergic neurons was suggested to be involved in maternal reward and motivation [63]. The upregulation of CHAT and SLC5A7 in breeding zebra finch suggests increased acetylcholine level in this period, which could contribute to the elevated motivation to care.

Cholinergic neurons are not present in the hypothalamus but are abundant in the medial septum, which was included in our dissections. Therefore, alterations in cholinergic neurons may take place in the medial septum and not in the hypothalamus.

#### 3.3.4. Brain Function of Thyroid Hormones

There is a well-documented decline in thyroid hormone levels related to caring for nestlings in hen [64]. The level of tri-iodothyronine (T3) drops before hatching, while the level of thyroxine (T4) decreases within a week after hatching, and both remain low during the breeding period. We found a number of gene expression changes regarding the thyroid hormone system, which suggests a similar change in the zebra finch during breeding.

The CRYM (crystallin mu) gene encodes a crystallin protein that binds NADPH and regulates thyroid hormone action: it promotes T3 binding to thyroid hormone receptor-containing dimers [65]. The drop of T3 and T4 around hatching may reveal the reduced importance of CRYM during post-hatching care, which is consequent with the downregulation of CRYM in breeding pairs in our experiment.

The ALDH1A3 gene encodes aldehyde dehydrogenase (aldehyde dehydrogenase 1 family, member A3) that produces NADPH. Since CRYM is described as a NADPH-dependent binding protein of T3, the expression of these two genes might be interconnected; CRYM can only regulate T3 binding in the presence of NADPH, which is produced by the aldehyde dehydrogenase enzyme [65].

The SLCO1C1 gene encodes the protein solute carrier organic anion transporter family member 1C1, which is a transmembrane receptor mediating the uptake of thyroid hormones in the brain in a sodium independent way [66]. Its downregulation is consistent with a reduced level of thyroid hormones to transport into the brain.

The TG gene codes the thyroglobulin protein, which is the substrate for thyroxine and tri-iodothyronine synthesis. Interestingly, the expression of the TG gene has been recently reported in limbic and hypothalamic brain regions [67]. Although its function in the brain is not known, our present data suggest that it may be related to adaptation to parenting.

The downregulation of genes (CRYM, ALDH1A3, TG and SLCO1C1) related to thyroid function in the breeding group can be interpreted as upregulation in the paired group, meaning that thyroid hormone-related function is more relevant during pair bonding than parenting, which is in line with previous explanations [64,68]. The pair bond formation is the initial step towards parental behaviour, especially to egg laying and incubation, so the higher expression of these genes in the paired group compared to the breeding group may induce transition mechanisms for the next reproductive stages [68].

#### 3.3.5. Pro-Opiomelanocortin

The POMC gene (pro-opiomelanocortin) encodes a preproprotein that undergoes post-translational processes at eight potential cleavage sites based on the cellular need and type of tissue. The prohormone convertase enzyme can produce different biologically active peptides with different cellular functions in rodents [69] as well as in birds [70]. POMC neurons of the hypothalamus, located in the arcuate nucleus, decrease food intake and increase energy use by integrating adiposity and satiety signals from the hypothalamus and brainstem in rodents [71] as well as in avian species [72]. These neurons reduce food consumption by serving as homeostatic sensors of blood hormone content and projecting to several other hypothalamic centres involved in food intake regulation using the peptide product alpha-melanocyte stimulation hormone (α-MSH) as their peptide neurotransmitter. The downregulation of the POMC gene in the breeding group suggests a reverse effect: inhibition of these neurons may allow increased food intake and a reduced energy expenditure in parenting females, which is necessary for increased feeding of the nestlings by regurgitation as previously suggested in the ring dove [73]. The correlation analysis also supported the relation of POMC and food consumption, namely, the lower level of POMC (reduced energy use) resulted in increased time spent regurgitating food (without change in body mass).

The downregulation of the POMC gene may result in the decreased expression of all peptide products in the parenting group. Some POMC peptides (α-MSH and ß-endorphin) are also involved in promoting the sexual behaviour of female rats by acting in the medial preoptic area [74]. The downregulation of POMC suggests that it also participates in the reduced sexual behavioural activity of breeding females. On the other hand, the high expression level of POMC in the paired group may promote sexual receptivity and the sexual behaviour of female zebra finches to increase the willingness to mate.

#### 3.3.6. Neuro-Immunological Alterations

In the brain, genes related to immunological response are mostly expressed in microglial cells as they provide immunoprotection of neurons. Microglia also contribute to neuroplastic changes under physiological circumstances [75]. Since parenting includes profound behavioural changes, neuroplastic alterations in the brain are plausible, and have indeed been reported in rodent mothers. The network analysis suggested the most significant involvement of Interleukin-4 and -13. These structurally similar cytokines signal through the same type II interleukin-4 receptor [76]. It has been suggested that they drive microglial cells towards an anti-inflammatory phenotype [77]. As far as a functional role of microglia in mothers, the literature is scarce. A potentially relevant suggested function of microglia includes regulation of mood control [78], frequently altered in the postpartum period.

## 4. Materials and Methods

### 4.1. Animals

Sexually mature adult zebra finches were randomly selected from the population maintained at the Animal House of Eötvös Loránd University, Hungary. The captive population was established from domesticated stock from Bielefeld University, Bielefeld, Germany [79]. The individuals were identified by a numbered aluminium ring (Principle Kft., Újlengyel, Hungary). Food (a seed mixture, supplemental egg-food, and germinated seeds) and water were provided ad libitum. Males and females selected from the population for the experiment were housed in separate double-cages (100 × 30 × 35 cm). The birds were included in the study if pair bonding took place. For the breeding group, a wooden nest box (12 × 12 × 12 cm) was attached from outside as described previously [23]. Coconut fibres were provided every day as nest material. The nest boxes were checked every day after the beginning of incubation (the first time egg was found warm in the nest) to observe the day of hatching. Optimized conditions were maintained with constant temperature and humidity level—20–21 °C and 55–60%, respectively—and with a standard light: dark cycle of 14:10 h. Offspring of the sacrificed parent were further raised by the other parent and recruited to the stock population on PHD-35, i.e., well after becoming independent of parental provisioning.

### 4.2. Experimental Design

**Figure 5:**
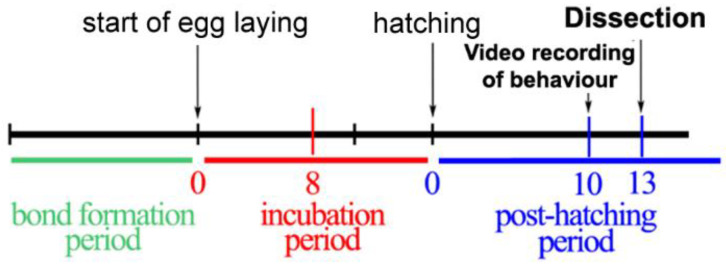
The time point of video recording and dissection in relation to the timeline of reproductive phases in the breeding group. The behaviour of the parents was recorded on post-hatching day 10 (PHD-10), while the dissection of their brain took place on PHD-13. The colours represent different stages of reproduction.

Breeding pairs and social pairs were the experimental and control groups, respectively. Breeding pairs were housed together until the dissection on PHD-13, while social pairs were housed together for at least two weeks after pair bonding. In the breeding group, nest boxes were checked every day after the beginning of incubation (first time eggs were found warm in the nest) to determine the day of hatching. Dissection of the septal–hypothalamic area took place on PHD-13, which was counted from the day when the first egg hatched in a given clutch [23]. Breeding parents were taking care of the offspring, while social pairs had neither nesting nor breeding opportunity. On PHD-13, at 14:00 p.m., the females were removed from the cage, sacrificed and their brains were dissected (Figure 5).

### 4.3. Video Recording of Parental Behaviour

The parental behaviour of the breeding pairs was recorded on PHD-10 for 3 h (from 10:00 a.m. to 13:00 p.m.) using small digital cameras with wide-angle (116° field of view) lenses (Mobius Action Cam, JooVuu Store, Greater Manchester, UK) placed on the top of the nest box, facing down. Previously, a piece of painted wood that resembled the real camera was mounted as a dummy camera, so birds were already habituated to the presence of it, and replacing with the real camera did not elicit neophobia. The camera stored video recordings on a microSD card. Video recordings were later behaviourally coded using Solomon Coder version 16.06.26 developed by András Péter [80].

### 4.4. Microdissection of Brain Samples

**Figure 6:**
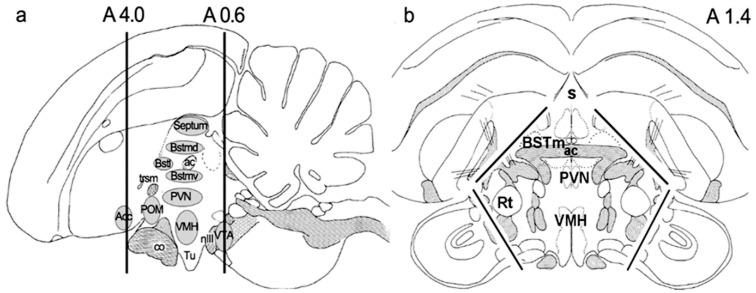
The schematic drawings of dissected hypothalamic–septal region: (**a**) sagittal, (**b**) coronal section. The anterior and posterior coordinates of the dissection of a coronal slide were A4.0 and A0.6. The black lines on (**b**) represent the included hypothalamic and septal regions with the removal of tectum and pallial structures.

For microdissection, 6 breeding females and 6 socially paired females were used. First, a 3.4 mm thick coronal section was cut with a razor blade in a brain coronal matrix (Alto Stainless Steel 0.5 mm Rat Brain Coronal 300–600 g) at anteroposterior coordinates 4.0 and 0.6 mm. These coordinates were determined using the stereotaxic atlas of the zebra finch [81]. Then, laterally and dorsally located brain regions, including the nidopallium (previously called neostriatum) and mesopallium (previously called hyperstriatum), were removed with a razor blade [82]. The remaining dissected brain tissue included the hypothalamus, the septum, and some parts of the adjacent medial striatum (Figure 6). The dissected samples were frozen immediately in 2-methylbutane on dry ice and stored at −80 °C until further usage.

### 4.5. RNA Sequencing

RNA extraction, library construction, and sequencing (Illumina Platform) were performed by BGI Tech solutions (Hong Kong) Co., Limited using a strand-specific library strategy with the UNG enzyme method (using UNG enzyme to digest second strand cDNA) and a sequencing strategy using the Hiseq platform PE101/PE151. Transcriptome strand-specific library construction was performed using the UNG enzyme method (dUTP method), in which RNA extraction, fragmentation, and the first strand cDNA synthesis pipeline were the same as the normal transcriptome; only the second strand cDNA’s synthesis was different (dUTP instead of dTTP). The dUTP method incorporates deoxy-UTP during the second strand cDNA synthesis and subsequently destructs the uridine-containing strand with UNG (Uracil-N-Glycosylase) in the sequencing library, allowing the identification of the orientation of transcripts.

#### 4.5.1. Library Construction

Total RNA was extracted followed by library construction consisting of the following steps: (1) DNase I digest to degrade double-stranded and single-stranded DNA in RNA samples. (2) mRNA isolation: Poly (A)-containing mRNA molecules were purified from total RNA using poly (T) oligo-attached magnetic beads. (3) mRNA was fragmented into small pieces using covaris. (4) cDNA synthesis was performed by first-strand cDNA generated using random hexamer-primed reverse transcription. This was followed by a second-strand cDNA synthesis and purification of cDNA. (5) The synthesized cDNA was subjected to end-repair and then was 3′ adenylated. Many rounds of PCR amplification were performed to enrich the purified cDNA template using PCR Primers. (6) After that, the short fragments were connected with adapters. Adapters were ligated to the ends of these 3′ adenylated cDNA fragments. The products of the ligation reaction were purified on TAE-agarose gel. (7) Library quality control was performed by validation with the Agilent Technologies 2100 Bio-analyzer and the ABI StepOnePlus Real-Time PCR System.

#### 4.5.2. Handling of Sequencing Data

The library was then sequenced using Illumina HiSeqTM4000. The original image data were transferred into sequence data via base calling, which is defined as raw data or raw reads and saved as FASTQ files. The raw sequencing data were deposited into the BioProject database on 21 February 2022 (http://www.ncbi.nlm.nih.gov/bioproject/808854, accessed on 21 February 2022). The Submission ID is SUB11104013 while the BioProject ID is PRJNA808854.

#### 4.5.3. Data Pre-Processing and Alignment

The quality of raw fastq files obtained from BGI were checked using FASTQC tool (http://www.bioinformatics.babraham.ac.uk/projects/fastqc/, accessed on 21 February 2022). The low-quality reads and adapter sequences were then trimmed using Trimmomatic [83]. The pre-processed high-quality reads were then aligned using RNA-Seq aligner STAR-2.6.0a [84] against the reference genome *Taeniopygia guttata* (zebra finch) downloaded from the Ensemble database (ftp://ftp.ensembl.org/pub/release-95/fasta/taeniopygia_guttata, accessed on 21 February 2022). The quantification and abundance estimation of transcripts were carried out using String tie tool v1.3.4 [85] from the paired-end RNA-seq dataset. Transcripts whose length was shorter than 500 bp were filtered out. Stringtie-merge was used to merge all the transcript files together to regenerate a reference gene model.

#### 4.5.4. Differential Gene Expression Analysis

Cuffdiff-v2.2.1 and cummeRbund-v2.24.0 R package from Cufflinks (http://cufflinks.cbcb.umd.edu/, accessed on 21 February 2022) were used for differential expression analysis and visualization, respectively. Cuffdiff takes the aligned reads from two or more conditions and reports genes and transcripts that are differentially expressed using a rigorous statistical analysis.

#### 4.5.5. Gene Set Enrichment Analysis

Gene set enrichment analysis was performed using g:Profiler [86] to identify Gene Set Enrichments and pathways using the method of Holm and Benjamini and Hochberg. The mammalian ontologies were used to account for the lack of sufficient data in zebra finch.

### 4.6. Real-Time PCR Validation

RNA samples returned from BGI company as leftovers following sequencing were used for the PCR validation in the CFX96 Real-time System of Bio-Rad. Dilution of the RNA to 2 µg/µL was followed by treatment with Amplification Grade DNase I (Invitrogen, Thermo Fisher Scientific, Waltham, MA, USA). Then, the cDNA was synthesized with the Superscript II reverse transcription kit (Invitrogen) according to the manufacturer’s instructions. Reverse transcription was followed by 10-fold dilution in RNA and DNA free water, and 2.5 µL of the diluted cDNA was used to perform PCR reactions using SYBR green dye (Sigma, St Louis, MO, USA). For the PCR reaction, iTaq DNA polymerase (Bio-Rad Laboratories, Hercules, CA, USA) was used to perform the PCR reactions. The PCR reactions were performed in a total volume of 12.5 µL based on the following protocol: 95 °C for 3 min, and then 95 °C 0:10 min, 60 °C 0:30 min, 72 °C 1:00 min for 41 cycles. Fluorescence was measured in each cycle at a temperature determined based on the melting temperature of the given PCR product (between 83 and 94 °C). After, the PCR reactions cycle threshold (C_T_) values were obtained from the linear region of the baseline-adjusted amplification curves, as described previously [87,88]. To establish quantitative differences between the examined gene expressions, the 2^−ΔΔ*C*T^ method was used. There were three housekeeping genes used: Glycerinaldehyde-3-phosphate dehydrogenase (GAPDH), Tubulin beta 4 (TUBB4B), Hypoxanthine phosphoribosyl-transferase 1 (HPRT1). None of them showed altered levels between the 2 groups. Their average was used for the 2^−ΔΔ*C*T^ calculation in each sample. Due to the small amount of RNA left after sequencing, only 2 genes were involved in the validation procedure: CRYM and POMC. The primers listed in Table 5 were used for PCR reactions at a final concentration of 300 nM.

### 4.7. Statistical Analysis

The statistical analysis of RT-PCR and the correlation analysis of PCR and behavioural data were performed using IBM SPSS Statistics version 23. In the correlation analysis, the threshold cycle values (CT) of the PCR were used for each individual to investigate whether the expression level of genes predicts the performed behaviour of the individuals. In the correlation analysis of the sequencing and PCR data, the FC values were used for the analysis.

### 4.8. Protein–Protein Interaction Analysis of Differentially Expressed Genes

To evaluate interactions among up- and downregulated genes, protein–protein interactions of DEGs were identified using the STRING online database (version 11.5) [89]. The Markov Cluster Algorithm (MCL) was used for clustering the proteins that were displayed in the network. The minimum required interaction score was 0.4 and the inflation factor was set at 2.5. To determine the function of genes in each cluster, the STRING online database was used for the functional annotation of clusters with GO terms [90], Reactome [91], KEGG [92] and Wiki Pathways [93]. Tissue expression of genes was obtained from the TISSUES web resource [94]. Functional annotation terms were considered significantly enriched with an FDR < 0.05.

## 5. Conclusions

A similar gene expression study in zebra finch or any other avian species has not been performed previously. Although some individual genes have been addressed in birds as far as caring behaviour [95,96,97], we often had to refer to mammalian functions of the DEGs

Any gene whose expression level is altered in the post-hatching period is a candidate to play a role in maternal adaptation. Since zebra finches do not lactate [22], the likely function of the DEGs is in the control of caring behaviours. While there are different types of specific caring behaviours, such as feeding the nestlings, nest building, etc., the DEGs could also be involved in general motivation changes towards the offspring characteristic of this period. Since we have not performed further functional studies, our own data provide essentially no information on which aspect of caring behaviour a particular gene is involved in. Still, the genes themselves had sometimes previously established functions, which allowed conclusions to be drawn on their possible role in the post-hatching period, too. Thus, this study first suggested the role of the dopaminergic system in parental care in birds; also, the evidence of the upregulation of the cholinergic system is very strong based on changes in several related genes. In turn, a reduced activity of the thyroid hormones as well as pro-opiomelanocortin and some immune processes during breeding was first suggested by our results.

## Figures and Tables

**Table 1:** The percentage of mapped reads for each sample.

Sample_Name	Raw Reads (mlns)	Uniquely Mapped Reads Number (mlns)	Uniquely Mapped Reads %
Breeding 1	21.6	18.1	83.8%
Breeding 2	21.2	17.7	83.6%
Breeding 3	21.6	18.0	83.6%
Breeding 4	21.1	17.7	83.9%
Breeding 5	21.5	18.0	83.9%
Breeding 6	21.7	18.0	82.9%
Paired control 1	21.2	17.9	84.4%
Paired control 2	21.5	18.2	84.4%
Paired control 3	21.2	18.0	84.8%
Paired control 4	21.6	18.2	84.2%
Paired control 5	21.4	18.1	84.2%
Paired control 6	21.5	18.2	84.3%

**Table 2:** The name and code of the 31 well-annotated DEGs used in further analyses.

Gene Name	Ensemble Gene ID	Protein Name	Log2FC	*p*-Value
SLC6A2	ENSTGUG00000007218	Solute carrier family 6 member 2 (sodium dependent noradrenalin transporter)	3.366	<0.001
TH	ENSTGUG00000009295	Tyrosine hydroxylase	2.550	<0.001
TPH2	ENSTGUG00000007300	Tryptophan hydroxylase 2	2.400	<0.001
CHAT	ENSTGUG00000005959	Choline-O-acetyltransferase	1.979	<0.001
SLC10A4	ENSTGUG00000008069	Solute carrier family 10 member 4 (acidification of synaptic vesicles containing monoamines)	1.896	<0.001
SLC5A7	ENSTGUG00000009694	Solute carrier family 5 member 7 (presynaptic sodium-dependent high-affinity choline transporter 1)	1.656	0.044
MIRLET7D, tgu-let-7a-3, tgu-let-7f	ENSTGUG00000017619ENSTGUG00000017688ENSTGUG00000017729	NA	1.525	<0.001
TG	ENSTGUG00000012562	Thyroglobulin	−1.528	0.003
ABCA10	ENSTGUG00000003994	ATP Binding Cassette Subfamily A Member 10	−1.560	0.007
ANLN	ENSTGUG00000005487	Anillin Actin Binding Protein	−1.571	<0.001
PMP2	ENSTGUG00000011663	Peripheral Myelin Protein 2	−1.585	0.002
ALDH1A3	ENSTGUG00000008854	Aldehyde dehydrogenase 1 family, member A3	−1.649	0.005
CILP	ENSTGUG00000009096	Cartilage Intermediate Layer Protein	−1.677	0.003
JAK3	ENSTGUG00000017135	Janus kinase 3	−1.697	0.032
OLFML1	ENSTGUG00000005163	Olfactomedin Like 1	−1.704	0.035
CRYM	ENSTGUG00000009095	Crystallin mu	−1.741	0.003
C1S	ENSTGUG00000013265	Complement C1s	−1.783	0.001
VIM	ENSTGUG00000001298	Vimentin	−1.821	<0.001
CLDN1	ENSTGUG00000009337	Claudin 1	−1.824	0.037
SEMA3B	ENSTGUG00000006529	Semaphorin-3B	−1.949	0.005
ZIC3	ENSTGUG00000002155	Zic family member 3	−1.951	0.015
MSX1	ENSTGUG00000009947	Msh homeobox 1	−2.056	0.003
SOCS3	ENSTGUG00000003345	Suppressor of cytokine signalling 3	−2.121	0.018
VILL	ENSTGUG00000000377	Villin Like protein	−2.166	0.036
RBPMS2	ENSTGUG00000004722	RNA Binding Protein, MRNA Processing Factor 2	−2.261	0.006
ADGRD2	ENSTGUG00000007153ENSTGUG00000007166	Adhesion G Protein-Coupled Receptor D2	−2.275	<0.001
LOXL2	ENSTGUG00000017286	Lysyl oxidase homolog 2	−2.503	0.005
HDC	ENSTGUG00000007569	Histidine decarboxylase	−2.601	0.002
SLCO1C1	ENSTGUG00000012325	Solute carrier organic anion transporter family member 1C1	−2.672	<0.001
TMEM255B	ENSTGUG00000009312	Transmembrane protein 255B	−2.812	<0.001
POMC	ENSTGUG00000017017	Pro-opiomelanocortin	−5.168	<0.001

The genes are arranged in order according to their fold-change values (log2FC). The colours and intensity represent the gene expression level: red indicates upregulated and blue downregulated genes in the parental group. Light red and blue colours represent genes with abs(log2FC) between 2 and 5. Not applicable (NA) indicates the lack of available protein name.

**Table 3:** Significant GO terms among upregulated and downregulated genes.

**Upregulated**						
**Source**	**Term_Name**	**Term Id**	**Adjusted** ***p*** **Value**	**Term** **Size**	**Query** **Size**	**Intersection** **Size**
KEGG	Tryptophan metabolism	KEGG:00380	0.026	32	9	1
KEGG	Tyrosine metabolism	KEGG:00350	0.026	23	9	1
GO:CC	Synapse	GO:0045202	0.003	584	9	4
GO:CC	Integral component of membrane	GO:0016021	0.019	3080	9	5
GO:MF	Neurotransmitter transmembrane transporter activity	GO:0005326	0.001	32	9	2
GO:MF	Monooxygenase activity	GO:0004497	0.002	66	9	2
GO:BP	Regulation of neurotransmitter levels	GO:0001505	0.002	166	9	3
GO:BP	Transmembrane transport	GO:0055085	0.006	986	9	4
**Downregulated**						
**Source**	**Term_Name**	**Term Id**	**Adjusted** ***p*** **Value**	**Term** **Size**	**Query** **Size**	**Intersection** **Size**
KEGG	Neuroactive ligand-receptor interaction	KEGG:04080	0.048	233	36	3
KEGG	Melanogenesis	KEGG:04916	0.048	70	36	2
GO:BP	Mesenchymal cell differentiation	GO:0048762	0.030	129	36	4
GO:BP	Thyroid hormone transport	GO:0070327	0.030	6	36	2

**Table 4:** Up- and downregulated genes contributing to KEGG pathway and enriched gene sets.

**Upregulated Genes**		
**Ensemble ID**	**Gene Names**	**Protein Names**
ENSTGUG00000007300	TPH2	Tryptophan hydroxylase 2
ENSTGUG00000009295	TH	Tyrosine hydroxylase
ENSTGUG00000005959	CHAT	Choline-O-acetyl transferase
ENSTGUG00000011058	SLC18A2	Solute carrier family 18 member A2 (synaptic vesicular monamine transporter)
ENSTGUG00000009694	SLC5A7	Choline:sodium symporter (presynaptic sodium-dependent high-affinity choline transporter 1)
ENSTGUG00000005989	SLC18A3	Solute carrier family 18 member A3 (synaptic vesicular acetylcholine transporter)
ENSTGUG00000007218	SLC6A2	Monoamine transmembrane transporter
ENSTGUG00000008069	SLC10A4	Solute carrier family 10 member A4 (acidification of synaptic vesicles containing monoamines)
**Downregulated Genes**		
**Ensemble ID**	**Gene Names**	**Protein Names**
ENSTGUG00000017017	POMC	Pro-opiomelanocortin
ENSTGUG00000002625	EDNRA	Endothelin receptor
ENSTGUG00000013478	P2RY2	Purinergic receptor P2Y2
ENSTGUG00000010805	TCF7L2	RNA polymerase II proximal promoter sequence-specific DNA binding protein (Transcription factor 7 like 2)
ENSTGUG00000009947	MSX1	RNA polymerase II-specific DNA-binding transcription activator
ENSTGUG00000009095	CRYM	Crystallin mu
ENSTGUG00000012325	SLCO1C1	Thyroid hormone transporter

Table 4 lists the genes, which changed significantly based on *p*-value and also contributed to the significantly altered pathways. Some of them are DEGs because their fold change reached the limit (log2FC value was ±1.5); others are not DEGs because of their smaller fold change.

**Table 5:** Primers used for PCR validation. The three housekeeping genes are highlighted.

Name of Genes	Gene Symbol	NCBI Gene ID	Forward Primer	Range (bp)	Reverse Primer	Range (bp)
Actin beta	**ACTB**	751978	CGTGCTGTCTTCCCATCCAT	82–101	CTCTCTGTTGGCTTTGGGGT	351–370
Crystallin mu	CRYM	100229846	AGGACTCCTCTGTGCCTTCT	206–225	CTTCACTGCCCTCTCCTTGG	480–499
Glycerinaldehyde-3-phosphate dehydrogenase	**GAPDH**	100190636	GAGGGTAGTGAAGGCTGCTG	795–814	AGAGCTAAGCGGTGGTGAAC	1113–1132
Hypoxanthine phosphoribosyl-transferase 1	**HPRT1**	100231349	GTGTGATCAGTGAGACGGGG	590–609	CAAACAGCACAACCCAACCA	907–926
Pro-opiomelanocortin	POMC	-	TACGTCATGAGCCACTTCCG	97–116	CCTCATCCTCCTCCTCCTCC	394–413

## Data Availability

The data presented in this study are available on request from the corresponding author.
